# Monitoring Sound and Its Perception during the Lockdown and De-Escalation of COVID-19 Pandemic: A Spanish Study

**DOI:** 10.3390/ijerph18073392

**Published:** 2021-03-25

**Authors:** María Dolores Redel-Macías, Pilar Aparicio-Martinez, Sara Pinzi, Pedro Arezes, Antonio José Cubero-Atienza

**Affiliations:** 1 Department of Rural Engineering, EPS, Edificio Leonardo da Vinci, Campus de Rabanales, Universidad de Córdoba, 14071 Córdoba, Spain; ir1cuata@uco.es; 2 Department of Nursing, Pharmacology and Physiotherapy, Faculty of Medicine and Nursing, Universidad de Córdoba, 14014 Córdoba, Spain; n32apmap@uco.es; 3 Department of Physical Chemistry and Applied Thermodynamics, EPS, Edificio Leonardo da Vinci, Campus de Rabanales, Universidad de Córdoba, 14071 Córdoba, Spain; qf1pinps@uco.es; 4 ALGORITMI Centre, School of Engineering of the University of Minho, 4800-058 Guimarães, Portugal; parezes@dps.uminho.pt

**Keywords:** COVID-19, noise, sound quality

## Abstract

The lockdown measures in Spain due to the SARS-CoV-2 or COVID-19 (Coronavirus disease 2019) pandemic from 13 March to 21 June 2020 had extensive social and environmental implications. This study aims to understand how the measures of lockdown have influenced noise levels, as well as people’s perception of sound quality before and after lockdown, including de-escalation. For this purpose, an online survey was carried out. Moreover, the noise linked to the Global Positioning System (GPS) position of each individual respondent was recorded aiming to correlate the noise level with the result of the survey. An average reduction of over 30 dB was observed compared with the sound pressure level before lockdown. Furthermore, it was found that the loudness parameter, together with the overall level, increased as the country started relaxing restrictions. Additionally, results showed that the perception of noise quality changed depending on the phase of de-escalation (*p* < 0.01), the type of property (*p* < 0.05), and the outside noise (*p* < 0.01). Moreover, noise annoyance was determined considering age (*p* < 0.01), gender (*p* < 0.05), type of property (*p* < 0.001), and home refurbishment (*p* < 0.05). It may be concluded that the most important measure to decrease noise levels is the reduction of traffic noise, through using eco-friendly public transportation or bicycles and limiting nightlife hours.

## 1. Introduction

According to the World Health Organization, more than 100 million people in Europe suffer from the effects of noise [1]. Although noise as a pollutant is usually underestimated, it is a significant risk factor and has serious consequences on people’s health from physical disability, such as hearing impairment [1], to more life threating effects, such as arterial hypertension, ischemic heart diseases, strokes [2,3], depression, pneumonia, chronic obstructive pulmonary disease, and decreases the response of immune system [4,5].

Different studies have indicated how severe noise exposure increases the risk of premature deaths and mortality rate [2,4,5]. In this sense, Thacher et al. [6] showed that long-term exposure to noise resulted in an increase in the probability of dying from cardiovascular, pulmonary and immunological diseases by eight percent. Additionally, this study manifested how lower levels of income, education levels or living alone were linked to the negative effect of this risk factor [6]. Steve et al. [7], though an animal model, indicated how noise exposure provoked an oxidative stress reaction resulting in epithelial modifications, hypertension, and calcium changes in both cardiac and renal structures. Another study, focused on the relationship between road traffic noise and health effects in an urban area with one of the highest rates of population in the country, indicated how noise levels exceeded the local and international limits during the whole day [8]. This study reported how the residents considered that the noise was worse and 19% of them had difficulties sleeping and 19% had stress due to road traffic. Regarding these biological modifications and posterior diseases, a report published by the European Environmental Agency [9] reported how noise can cause malignant cell growth, resulting in cancers [3,10,11,12,13]. Furthermore, other studies have hypothesized that psychological stress due to noise exposure could aggravate respiratory illnesses already present or resulting from excessive noise [3]. Psychological illnesses, such as stress and annoyance, can be caused by exposure to noise, as well as serious mental health problems, such as depression and anxiety [3,14]. Other research studies have also studied more indirect associations between living in noisy areas and health. For example, sleep disturbances due to traffic noise which could induce physical inactivity [15,16], and transportation noise which could worsen healthy lifestyle factors, resulting in smoking, alcohol consumption or medication intake have also been investigated [16]. Despite the results, most studies focused on the perception or opinion of people focused on night periods, noise caused by neighbors and did not include the living conditions, such as antiquity of the building [8].

The Environmental Noise Guidelines for the European Region established that the burden of disease is of over 61,000 years for ischemic heart disease, 45,000 years for cognitive impairment in children, 903,000 years for sleep disturbance, 22,000 years for tinnitus and 654,000 years for annoyance [1]. Consequently, nearly one million healthy years of life are wasted every year from traffic-related environmental noise in western Europe. Sleep disturbance and annoyance, mostly related to road traffic noise, constitute the bulk of this burden. Available assessments place the burden of disease from environmental noise as the second highest after air pollution [1,17,18].

Moreover, the impact of environmental noise exposure not only affects humans but also animals. Research has highlighted how exposure to noise provoke an increase in infertility [16,19], modifying the reproductive processes, from reproductive physiology and development to sexual selection and parental care [20]. These results in animals and experiments with animal models seemed to further show the effect of noise in human or animal health.

## 2. Pandemic and Noise

Due to the COVID-19 pandemic, the governments of different countries, including China, Italy, Spain and France, declared lockdowns to limit social contact with the objective of flattening the epidemic curve. In Spain, for example, this measure started on the 14th of March by closing schools and many other activities and finished on the 21st of June. During this period, the government tried to limit contact between persons and citizens could only circulate the premises of their homes to acquire food or necessities. Other than that, travelling throughout the national area was limited for work/health reasons. On average, lockdown was carried out in 200 countries between 25 and 30 days. The number of COVID-19 cases was minimized with this measure. However, lockdown not only reduced the prevalence of COVID-19 but also entailed the improvement of environment pollution and air quality. Air pollution is one of the principal problems in recent decades with a high impact on human health and the environment. There are many sources of pollution varying from engine combustion of automobiles to industrial activities [21]. In these conditions of lockdown due to COVID-19, many newspapers and mass media have reported how, in major global cities, the level of pollution has dropped [22,23]. Some studies have focused on evaluating the environmental impact, especially air quality, of these restrictions. In the United Kingdom [24], NO_2_ levels were reduced over 60% compared with the same period in 2019. In New Delhi, a reduction in PM2.5 from 23rd March to 13th April was found compared to the same period in 2019 [25]. A higher reduction in NO_2_ emissions was recorded in New York (USA), as these were about 30% lower than the monthly average from 2015 to 2019 [26]. The European Space Agency, through measurements carried out by the Sentinel -5P satellite, showed reductions in measured NO_2_ levels over cities in Asia and Europe between January and February, of about 40–50% compared to this period in 2019 [26]. Additionally, CO and SO_2_ levels dropped in the city of Milan by an average of 57.6% and 25.4%, respectively [26].

Noise pollution also decreased during lockdown because road and rail traffic and industrial activities were reduced. However, sparse research about the impact of these restrictions on noise emissions has been reported. Aletta et al. [27] assessed the urban sound environment during lockdown using short-term acoustic measurements at 11 different locations in London. They found an average reduction of 5.4 dB(A) (LAeq), although the degree of reduction varied depending on the location from 10.7 to 1.2 dB(A). This suggested that the decrease in environmental noise relied on the urban context, so the authors proposed as future work to consider perceptual aspects. A reduction in environmental noise has occurred in other parts of the world such as New Delhi (India), where noise levels decreased to under 65 dB(A) [28]. In Stockholm (Sweden), the noise level reductions for lockdown were compared to those observed before lockdown, for the period ranging from mid-April 2019 to the end of June 2020 [29]. A maximum peak drop of about 4 dB(A) was found in April 2020, and a decrease of between 0.5 and 2 dB(A) was observed in June 2020 compared to the same periods in 2019. Therefore, the authors concluded that based on this trend, noise levels would return once lockdown was finished. Asensio et al. [30] also analyzed the reduction in noise pollution in Madrid (Spain) from March to June 2020. This research was carried out using a monitoring network of the sound level meters placed in different locations around this city. The authors found that the decrease in the sound level ranged from 4 to 6 dB(A) for the different periods during the day: Ld (noise level during the day from 7 a.m. until 7 p.m.), Le (noise level during evening from 7 p.m. until 11 p.m.) and Ln (noise level during the night from 11 p.m. until 7 a.m.).

Although the environmental noise level significantly decreased during lockdown, neighbors’ noise became more noticeable since people had to spend more time at home. Hence, neighbors’ noise has become one of the main problems among public complaints more and more often. For example, the Royal Borough of Greenwich has developed a mobile Noise App with the aim of giving users the chance to report and submit their own recordings of noise [31]. The reduction in noise has been such that even research on seismic activity has been able to clearly separate anthropic vs. natural components, which are not easily distinguishable [32]. Another effect that has been possible to observe is how animals may start wandering more freely across the oceans and streets, mainly due to the reduction in the vessel and road traffic and noise pollution levels [33]. These disturbances and previous opinions of people regarding noise are linked to beliefs about the external environment, changing accordingly to factors such as being inside their residence for more time [34]. The perception changes accordingly to people’s previous experiences, the period of the survey, age, sex, physical or emotional distress, and different forms of quantitative measures or data, such as the example of placebo effect [35,36,37]. In this sense, different studies in the health field have indicated the bias of survey opinions after a period of time. Based on previous statements and the main factor that COVID-19 is for perspectives [34,38], the hypothesis (H1) was that the data would show a significant decrease in noise that would not be in complete sync with people’s perception of the disturbance of noise.

Therefore, the main objective of the present study is to assess the impact of lockdown on noise pollution and on sound quality throughout all de-escalation phases and during lockdown in Spain, considering the more significative social and personal variables and comparing quantitative direct measures and people’s perception. In this sense, this study provides a photoacoustic perspective of the historic event and the perception of people to the change in global noise during lockdown.

## 3. Materials and Methods

### 3.1. De-Escalation Phases in Spain

**Figure 1:**
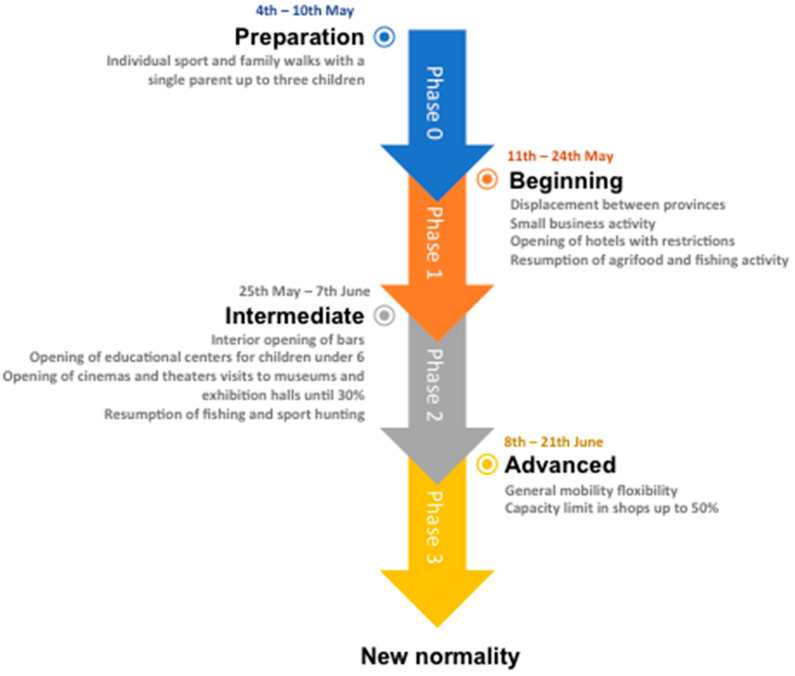
Phases of de-escalation.

After COVID-19 was first identified in Wuhan, China, it rapidly spread, starting a global pandemic. While there was no vaccine, the measures arranged to contain the spread of COVID-19 were national lockdowns and quarantines [39]. In Spain, lockdown started on the 16th of March and finished on the 21st of June, although until the return to normality, there were different phases of de-escalation (see Figure 1). It is important to mention that these phases happened on different timelines for several provinces in Spain. The de-escalation consisted of four phases where, at the beginning, all stores were closed except for supermarkets and essential needs providers such as pharmacies. Primary schools, high schools and universities were closed, and online classes were effectively implanted. In fact, these remained closed for the rest of the semester. Moreover, telework was established except for justified practitioners such as physicians and other healthcare-related providing workers, nurses, pharmacists, as well as other essential workers, among others. During the quarantines, the strictest measures were implemented, as citizens were required to stay at home and neither walks nor outdoor sports were allowed. The province borders were also closed. These restrictions were kept until the 10th of May (phase 0) and from then on, walks and outdoors sports were allowed although with some limitations because a schedule was established depending on the age of the population. From the 11th until the 24th of May (phase 1), travel between provinces was permitted, small businesses were allowed to open as were hotels, though with restrictions. In phase 2 in Córdoba (from the 25th of May until the 7th of June), education centers for children under 6 years old started opening. Cinemas and theaters, museums, and exhibition halls with a reduction in capacity of up to 30% also started opening and bars could make use of their interior spaces, provided the safety distance between people was observed. The last phase (from the 8th until the 21st of June), there was mobility between provinces without restrictions and the capability of the stores was increased up to 50%, since these were closed in the previous phases and lockdown. Finally, lockdown was concluded by the government on the 21st of June and the responsibilities of the measures of control were given to the Governments of Autonomous Communities.

### 3.2. Questionnaires

Two online questionnaires were developed by Aula de Software Libre of University of Córdoba using the open-source software LimeSurvey GmbH version 4.2.0 (Carten Schmitz, Hamburg, Germany). Both questionnaires (in Spanish) were possible to fill in using mobile phones through these links: https://encuestas.webapps.uco.es/index.php/276959?lang=es (accessed on 24 March 2021) for the first questionnaire and https://encuestas.webapps.uco.es/index.php/646179?lang=es (accessed on 24 March 2021) for the second one. The initial questionnaire was aimed at the first phase of lockdown and the second one was more related to the rest of the phases of the de-escalation. The initial questionnaire had 15 questions about demographic factors (age, gender and education) and situational factors (how the respondents were experiencing confinement: alone, with one’s partner or family, university accommodation; kind of housing: flat without balcony, house, flat with balcony, shared flat; ownership of the property; age of the building; insulation reforms and the kind of remodeling). Additionally, the geolocation was registered, and this was performed automatically through the Global Position System (GPS) coordinates from the mobile device. After these questions, the initial questionnaire was divided into two sections: one regarding annoyance prior to lockdown and the second part related to the annoyance during lockdown, although repeated questions were asked in both sections. Respondents had to quantify (on a Likert scale of 5 levels) their perception of different sources of noise, such as noise from traffic, from neighbors, from nature or from bars and supermarkets. Finally, exterior noise was recorded by the survey respondents (see Appendix A). The second questionnaire was proposed with the aim to compare noise perception during the different phases of the de-escalation (Appendix A). The first question of the second questionnaire was if this was the first time that the respondent filled in the questionnaire. If the response was “No”, the respondents were directed towards the second questionnaire; otherwise, the respondent was pointed to fill in the first questionnaire. Furthermore, the survey respondents were requested to record the noise with their mobiles and give theirs GPS position for both questionnaires.

### 3.3. Location of Respondents

**Figure 2:**
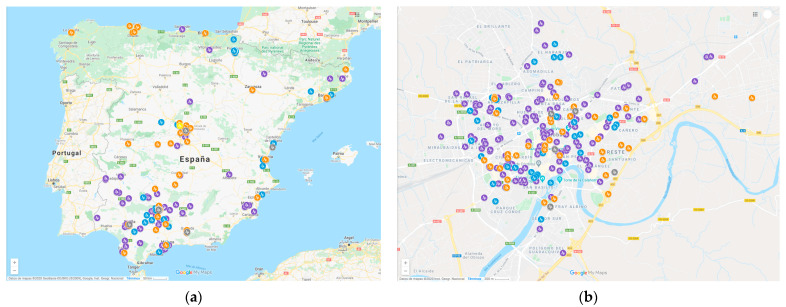
GPS positions of the results in Spain: (**a**) purple for lockdown, grey for phase 0, blue for phase 1, yellow for phase 2 and red for phase 3; (**b**) detail of results for questionnaires in Córdoba: purple for lockdown, grey for phase 0, blue for phase 1.

The GPS coordinates achieved from the filled out surveys were placed on a map using MyMaps from Google. The geolocation of the different answers of the questionnaires was mainly in Spain, achieving only a few responses in America, France, and Belgium, which corresponded to lockdown. The responses were obtained for different provinces in Spain, although, in general, these were in Córdoba (see Figure 2a,b). The dissemination of these surveys was carried out by the Scientific Communication from the University of Córdoba using different social media networks such as Twitter, YouTube, as well as other media such as newspapers, TV news, among others [40]. At the start of each new de-escalation phase, the second questionnaire was again spread on social media networks. A little more than 70% of the responses have been achieved from Córdoba as it is possible to see in Figure 2b.

### 3.4. Dataset

A total of 951 responses were collected for the first questionnaire, although 345 were incomplete responses due to participants not recording the external noise or not giving the GPS position correctly. For the second questionnaire, over 81 responses were obtained, with only 61 being completed. Moreover, once the dataset with the responses of registered noise was analyzed, it was observed that about 20% of the Waveform audio file format (WAV files) gave an erroneous simulation during the calculation of loudness.

The independent variables included the perception of noise in the previous stages and during the lockdown and phases. The dataset was saved in Excel version 17 (Microsoft, Redmond, WA, USA) and SPSS version 25 (IBM, Armonk, NY, USA).

Qualitative variables, such as gender, were codified using 0 for female and 1 for male. The option “not know, rather not to say or missing” was coded as 0 in all variables. The age was divided in ranks of age (under 20; 20–35; 36–50; 51–65; 66–80 and more than 80 years old) and was coded in ordinal ascending number starting with 1. The educational level was divided into three levels—without any studies, basic education, and university studies (including PhD)—being coded from 0 as lowest level and 2 as highest. The personal situation during lockdown was considered as “alone”, “with the family” or “in university residence”, being categorized from 1 as alone to 3 as in university residency. The type of house construction was “chalet” (coded as 1), “shared flat” (coded as 2), “flat with terrace” (coded as 3), or “flat without terrace” (coded as 4). If the living place is “rented”, it was coded as 1 or if “owned”, it was coded as 2. The year of construction of the building was “Before 1970” (coded as 1), “1970–1990” (coded as 2), “1990–2000” (coded as 3), or “after 2000” (coded as 4). The building reforms were coded as “1” for No and “2” for Yes. The perception of the level of noise was divided using a Likert scale from 1 as nothing to really severe as 5.

The dataset was analyzed using descriptive statistics and the relationships of the qualitative variables. Initially, data normalization was examined using the Kolmogorov–Smirnov test showing that the sample was not normalized (*p* < 0.001). Based on this result, the Chi-square U non-parametric test was used. Additionally, the Spearman correlation test was carried out to determine associations between the differences.

### 3.5. Recorded Noise

As mentioned above, the survey itself gave the possibility of making a recording of the external noise using the microphone of the mobile. The noise registered, when the survey respondents pressed the button in the survey, was stored in the database together with the survey response (see Appendix A). The sounds recorded were stored in WAV format file for further treatment using Testlab Siemens software (Siemens, Germany). Moreover, the Interlight S.L. company supplied long-term noise recorded in a specific location from Córdoba.

### 3.6. Noise Measurements

#### 3.6.1. Overall

For each registered noise, the overall noise level (dB) was calculated with the data collected for 10 s using the Simcenter Teslab software.

#### 3.6.2. Loudness

Knowing the decibel value of the sound, it is possible to give an idea about the amplitude of the sound, although this is not representative of the perceived loudness of the sound [41]. For this reason, as a psychoacoustic parameter related to the strong influence on sound quality, loudness is used, which gives a better characterization of how humans perceive a sound. The unit of the loudness used in this research was the “sone” and the standards for the calculation of sones are Stevens Mark VI and VII, which are available in Simcenter Teslab. The use of loudness conveys an idea of human perception of sound as the human hearing domain depends on the frequency. For example, a tone of 10 dB value at 100 Hz is inaudible, but at 2000 Hz it is audible; this means that the perceived loudness may be very different even though the sound pressure level in dB is the same for both tones. This metric was developed with a jury testing using persons to establish the curve of equal loudness, unlike dB which is based in a mathematical equation. For these reasons, the use of loudness is better than the use of dB values to correlate the perception of sound annoyance [41].

## 4. Findings

### 4.1. Environmental Noise Results

**Figure 3:**
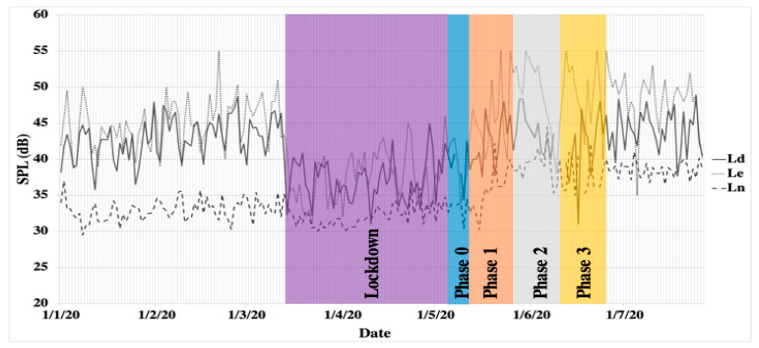
Long-term Sound Pressure Level (SPL) registered by Interlight S.L. L_d_: SPL for day period; L_e_: SPL for evening period; L_n_: SPL for night period.

Interlight S.L. provided long-term noise levels recorded with a measuring device in a critical part of the city that has high urban traffic between the months of January and July. Figure 3 shows the time series of the indicators defined in the European Environmental Noise Directive [42], L_day_ (L_d_), L_evening_ (L_e_), and L_nigth_ (L_n_), for lockdown and de-escalation phases. It is possible to observe that the Sound Pressure Level (SPL) decreased about 15 dBA during lockdown, especially in the evening time. Furthermore, it is possible to see how this decrease started on the 13th of March. It should be noted that, on the weekend that preceded the quarantine (13th until 15th of March), a call was made to all inhabitants requesting them to stay at home (see Figure 3). After phase 3, a slight decrease can be observed, which could be due to the summer holidays and the migration from the city center to countryside, reducing the traffic noise in the city. Even though there was a reduction in socioeconomic activities, the SPL after the de-escalation seems to lightly increase even further than before lockdown.

**Figure 4:**
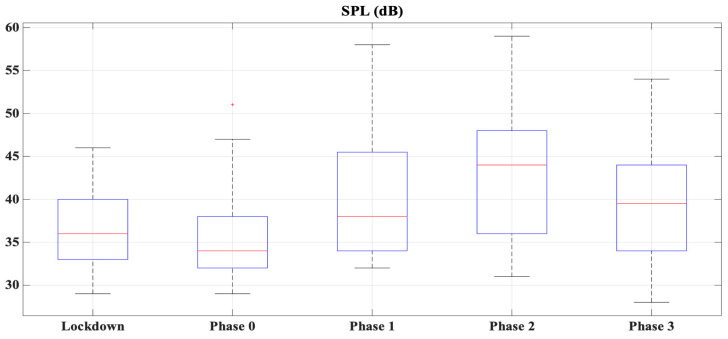
Boxplot of the Sound Pressure Level (SPL) during de-escalation in Ronda Marrubial Córdoba.

Regarding the sound quality (loudness) and the overall noise level registered by respondents with their mobile, it is possible to see from Figure 4 that both parameters increased in phase 2. This could be corroborated by Figure 4, which shows the Sound Pressure Level (SPL) during de-escalation in Ronda Marrubial in Córdoba. Thus, it is possible to observe the same trend for both figures, where the SPL is increased in phase 2 of the de-escalation.

**Figure 5:**
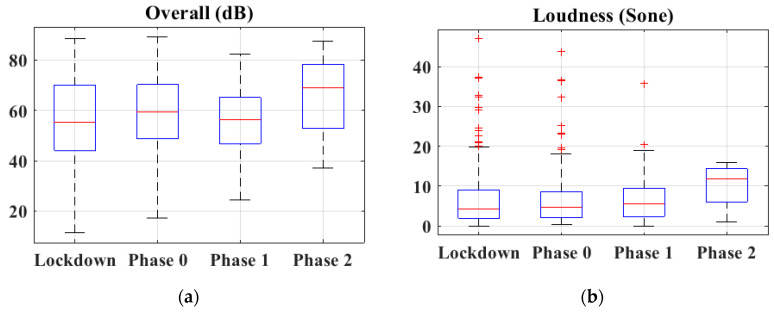
(**a**) Overall noise and (**b**) Loudness during lockdown and de-escalation.

In general terms, both loudness and overall noise seem to increase in phase 2, decreasing in phase 3 (see Figure 5). From Figure 4, it is possible to see how the noise increased in phase 1, reaching values similar or higher than before lockdown. The municipal bus company (Autobuses de Córdoba S.A.) supplied to the University of Córdoba the number of users during the months of June and July of 2019 and 2020. In 2020, for these months, the number of users was reduced to 51.33% and 36.12%, respectively.

**Figure 6:**
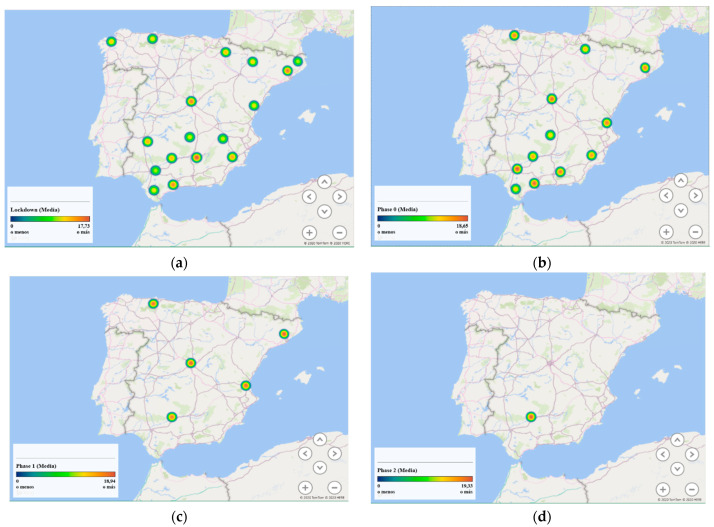
Bubble map of loudness during outbreak: (**a**) lockdown; (**b**) phase 0; (**c**) phase 1; (**d**) phase 2.

Figure 6 shows loudness for different provinces where the survey was responded. At the beginning of the outbreak, loudness was lower than in the rest of the phase due to the limitations in mobility and closed activities. When there were fewer restrictions on mobility and the opening of activities and shops started to take place, higher loudness can be observed. Therefore, phase 2 showed a higher loudness value compared to the other phases and lockdown. Regarding loudness in the different provinces, it is possible to see that during the lockdown, Jaen, Málaga, Madrid and Barcelona were the provinces with higher loudness values. This trend appears to hold in the rest of the phases, although due to the low number of responses achieved, it could only be appreciated for almost all phases in Córdoba.

**Figure 7:**
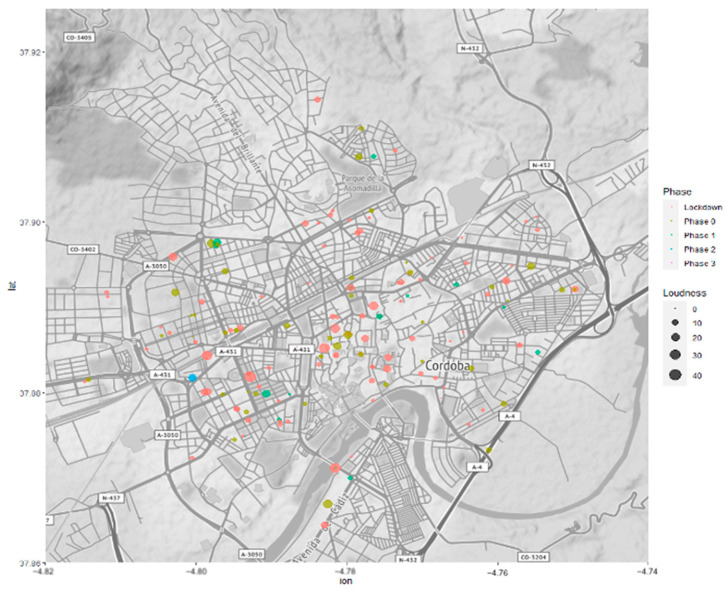
Bubble map of Loudness during outbreak: light red—lockdown; light green—phase 0; green—phase 1; blue—phase 2; red—phase 3.

A bubble map of loudness in Cordoba during lockdown and de-escalation is shown in Figure 7. The highest value of loudness corresponds to the hour of applauses in Spain (at 20:00). Besides this, the loudness stays below 20 sones, especially during lockdown.

### 4.2. Statistical Results from the Questionnaires

#### Sociodemographic Initial Analysis

The initial analysis of the data showed that most participants were female (59.3%) with ages ranging from 36 to 50 years old (41.7%), with a degree, master’s degree, or PhD (79.7%). Most of the participants lived with family members or partners (86.7%), in a flat with a terrace (52%), most of these being owned (78.6%), constructed after the 2000s (29.5%) and without any reforms (59.6%). Observing Table 1, which presents the correlations of the sociodemographic data, it is possible to see that a higher age was linked with having more cohabitants (*p* = 0.002), owning the property (*p* < 0.001) and carrying out modifications in the property (*p* < 0.001). Meanwhile, a higher educational level, such as a PhD, was related to having a house or independent flat (*p* = 0.015) and carrying out modifications in the property (*p* = 0.001).

### 4.3. Quality of Sound before the Lockdown

#### The Noise Annoyance before Lockdown Was Related to the Sociodemographic Data

From Table 2, it can be observed that there is a direct correlation between perception of sound before lockdown and age (*p* < 0.05): younger people, which tend to live in rental apartments without reforms, were the most conscious about the annoyance before quarantine. Noise perception in this age group could be related to the fact that older apartments without restructuring commonly had the worst level of isolation. The results depicted in Table 2 show that there was a relationship between gender and the perception of noise from nature before lockdown (*p* < 0.001), indicating that women had better perception of the noise from nature. Additionally, older people noticed more unpleasant noises from neighbors before lockdown (*p* < 0.001).

Regarding education level, it is possible to see how people with a lower education level were annoyed by nature (*p* < 0.001), which could be linked to the type of property (Table 2). In the case of property, ownership was inversely correlated to annoyance from outside, traffic, neighbors and businesses (*p* < 0.001), which implied that people renting properties found this kind of noise more irritating. Sharing property was linked to more irritation from the noise caused from outside (*p* < 0.001), traffic (*p* < 0.001), neighbors (*p* < 0.001) and different activities, such as bars or supermarkets (*p* < 0.001).

### 4.4. Sound Quality during the Lockdown

**Figure 8:**
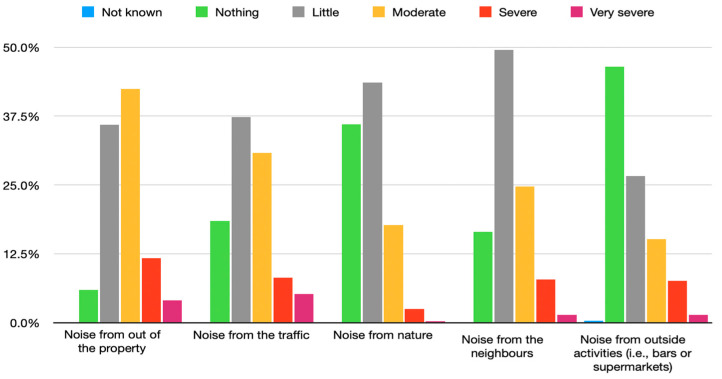
Frequencies regarding the description of the noise before lockdown.

**Figure 9:**
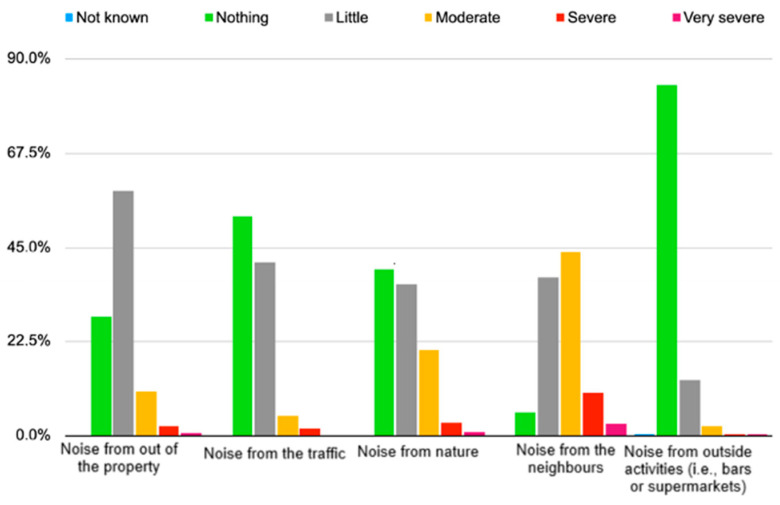
Frequencies regarding the description of the noise during lockdown.

The annoyance caused by noise before lockdown and during lockdown was also studied (Figure 8 and Figure 9). The perception of annoyance before and during lockdown was different, mainly being defined as “moderate” before lockdown and as “little annoyance” during lockdown. In fact, annoyance decreased by approximately 20% when comparing between before and during lockdown. In this case, annoyance during lockdown was mostly described as little (58.4%) or non-existing (28.3%). Disturbance from urban traffic noise also decreased over 25 percent, being mostly perceived during the lockdown as “peaceful” (52.4%). As for annoyance provoked by nature sounds, the results show that there was an increase in all descriptions of severe and very severe disturbance by 2.7 percent and 0.5 percent each when comparing the phase before lockdown and during lockdown. Disturbance caused by neighbors increased during lockdown by almost 20% (for “moderate”) when compared to before lockdown. This could be due to the increasing imposed time spent at home. Finally, annoyance caused by noise from outside activities (i.e., bars or supermarkets) seemed to be described more as little (26.6% before and 13.3% during the lockdown) or not an annoyance (46.6% before and 83.7% during the lockdown).

**Figure 10:**
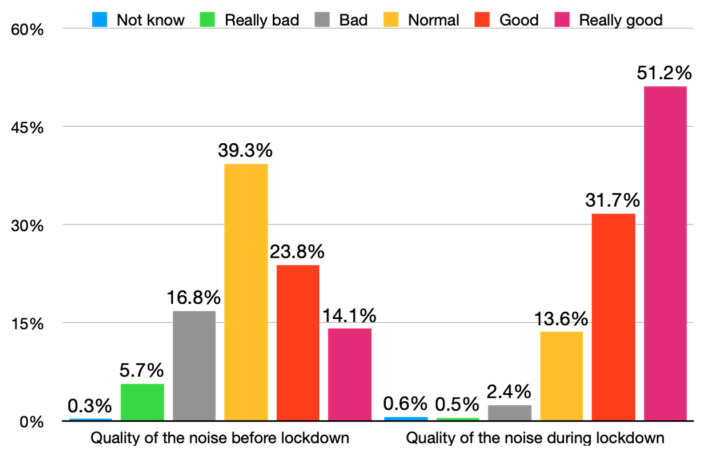
Sound quality before and during lockdown.

The global sound quality during lockdown improved drastically (see Figure 10). In this sense, Figure 10 shows that the sound quality of noise was worse prior to lockdown, the descriptions of “bad” (16.8%) or “good” (23.8%) being more common. During lockdown, noise quality was defined as “good” (31.7%) or “really good” (51.2%), representing an increase of a mean of 22.5 percent.

Additionally, the Spearman test showed a positive correlation regarding the quality of noise before and during lockdown (*p* < 0.001), indicating that the participants with a good perception before lockdown perceived the sound quality as really good during lockdown.

From Table 3, it is possible to observe annoyance during lockdown. Youngsters perceived the noise from outside as worse (*p* < 0.001), which could be because they usually spend more time outside of their home and in this situation, where they were forced to be confined, made them more conscious of the outside noise. People with lower educational levels had noticed noise from nature and neighbors to be not as good, which may be due to the positive correlation between educational level and kind of properties and recent modifications (Table 1). People who live in rented apartments were more conscious of annoyance due to noise from outside, nature, neighbors and bars and supermarkets, among others. The year of construction results as a decisive factor as newer buildings, probably with better acoustic isolation, provoked people to better perceive the noise from outside, traffic, neighbors, among others.

### 4.5. Sound Quality during Phase 0 and Phase 1

**Figure 11:**
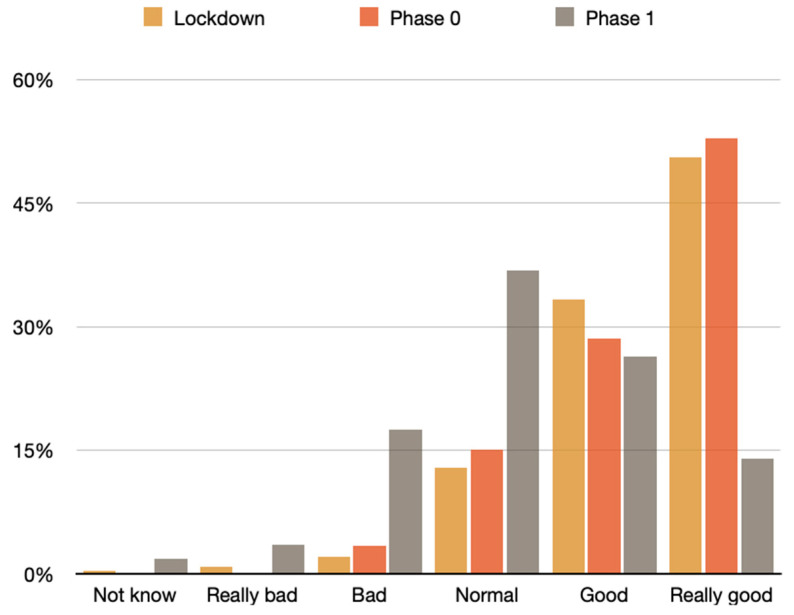
Sound quality during lockdown, phase 0 and phase 1.

Perceptions changed when the participants were in phase 0, which is a phase posterior to the lockdown, after the beginning of de-escalation. In this stage, the participants noted an increase in annoyance from noises from outside of the property by 5.5 percent, and from traffic by 5.9 percent. In contrast, the participants described a small reduction in annoyance caused by neighbors (1.2%); and nature (0.7%) (Figure 11).

The analysis of sound quality during lockdown and phase zero showed no significant differences between participants in phase 0 or 1 (*p* > 0.05). This could be due to the difference in loudness being very small. The sound quality during the phases, which was not linked between each phase (*p* > 0.05), seemed to improve at the same time as that of the perception of noise from outside (*p* < 0.001), traffic (*p* < 0.001), neighbors (*p* < 0.001), outside activities (*p* < 0.001), trains (*p* = 0.044) and airplanes *p* = 0.005) became worse. Moreover, sound quality during the phases was linked to a better opinion regarding the quality of noise perceived prior to the lockdown (*p* < 0.001). Moreover, just at the beginning of lockdown, noise levels dropped by almost 30 dB, which matches with the decrease in annoyance caused by nature, neighbors, or outside activities. The more common description during lockdown from annoyance was little trouble from noise outside (60.6%) and from nature (41.0%); and no annoyance caused by neighbors (45.8%), traffic (54.2%); exterior activities (84.3%) (i.e., bars or supermarkets), trains (90.0%) and airplanes (87.6%).

Sound quality was studied for lockdown and phase 0 (Figure 11), which showed that as participants were moving to each further de-escalation phase, the opinion regarding the quality before lockdown became worse (*p* < 0.001). In fact, further analysis of the three phases and the sound quality before the lockdown showed a significant negative link between the de-escalation and the quality of noise before lockdown (*p* < 0.001).

## 5. Discussion

The results regarding environment noise from the Interlight S.L. company showed a significant decrease caused by lockdown and a rapid posterior growth resulting from the de-escalation, increasing even further than before lockdown and despite the reduction in mobility. These results could be caused by the population’s perception of the use of public transport as a risk to their health, resulting in using individual vehicles, such as cars, or even due to the opening of bars and commercial activity. In this sense, it is interesting to note that the data from public transportation indicated a significant decrease in buses, which could confirm previous statements regarding the use of individual vehicles. Madrid’s Metro has also confirmed this trend, where the number of passengers dropped to almost 40% [43]. This increase in noise levels and decrease in using public transportation could be linked to the rapid growth of outsides exercise or fitness, which contrasted with the sedentary time and less physical activity caused by isolation and lockdown [44].

Levels from lockdown to de-escalation phases have shown some differences between cities, these being with higher levels for those with a more significant population and bigger peripheries. These results matched previous studies that indicated that noise levels were linked to city centers, the number of industrial companies, and the number of people inside the region [8,25,29,30]. Moreover, the data gathered by the company and the participants reflected how the quantitative data from specific or impartial companies and the population were symmetrical regarding the significant reduction in noise during lockdown, and posterior return to previous noise levels as the de-escalation moved further onwards. All this information showed that despite the initial noise reduction caused by lockdown, the levels of sound tend to maintain their usual standards for urban structures despite regulations or decreases in traffic. These results could be linked to people’s capacity to adapt to environmental discomfort and the need to feel previous normality before lockdown [45].

In sync with the quantitative data, the survey results indicated that the individual’s perception was mediated by the sociodemographic, individual, or living conditions, and furthermore, this perception was modified by the time or de-escalation, worsening as time set from lockdown and the opening of the cities was closer. The H1 has been partially obtained with all the results since it has indicated a significant decrease in noise. Still, in this case, people’s perceptions were cohesive, with the data being contradictory only in a few factors. These results are impressive since it could be argued that the perception of noise was going to be mediated by isolation, and the perspective be modifiable and less precise as time passed. Nevertheless, it could be argued that the lockdown and de-escalation have marked the perception of the population so much that the quantitative and qualitative results were cohesive. These results seemed to matched previous researchers that stated how mixed methods appeared to be a highly effective research approach when significant events happen, such as patient death [46].

Additionally, the participants’ perceptions were linked to individual or personal variables, such as gender, age, or educational level; the living conditions or building environment, such as the year of construction or type of property; and the personal opinion regarding the annoyance caused by different factors from neighbors to nature. These results corroborate the findings of a great deal of the previous works on annoyance, noise levels, individual perception, and living conditions [3,6,11,13]. However, other authors have not previously described all these results, linking only a few variables based on surveys [8,13].

The current study, as with any research, has some limitations. The survey data are based on people’s opinions with a transversal cut and not all the variables should be applied linked to other populations or in different set times. Additionally, because of the state and content of the survey, the participants were not asked about any health modifications, highlighting the positive effect of noise reduction. This could also be a bias factor since participants and the industries took these measurements during isolation. Due to the COVID-19 and lockdown, the exact measurements and data from GPS seemed to be limited to this exception in time. However, it could be used as an example of the decrease in noise and the rapid recovery of stock levels in a short period.

Despite the limitations, the current study presents an innovative perspective combining quantitative data, using exact measures and GPS, and a population’s perception regarding noise. This approach has not been made previously since more studies focused on establishing health problems and noise measures but did not include perception in different periods, including individuals’ and buildings’ factors, and possible originators of noise. Although they are country-specific to Spain and specifically the south, these results and data can be transferred to Europe as a universal research method.

## 6. Conclusions

This research has analyzed the impact of lockdown and the de-escalation phases on noise emissions due to the pandemic situation. Moreover, perceptions of sound and annoyance have been correlated with demographic and situational factors. The results have shown a reduction in sound pressure level during the confinement. However, it increased during the last phase of de-escalation in Córdoba. This reduction has been noted in the whole of Spain. In Córdoba, lower levels of noise were achieved during lockdown, observing an average decrease of about 10 dB for Ld and Le. This can be explained by the lack of activity and mobility reduction, which minimized the traffic noise. Additionally, it has been corroborated that there is a fear of taking public transport, so people tend to use their private vehicles.

Regarding noise annoyance, it is possible to affirm that sociodemographic factors, such as gender, age, type of property, among others, impact the perception of noise for respondents. The respondents’ age and gender, the type of property, educational level, the ownership of the property, and the year of construction seemed to play a vital role in noise annoyance before and during the lockdown’s de-escalation. For instance, younger people and individuals with the lowest educational level seemed to be more bothered by the noise from the outside, e.g., neighbors or nature, which appeared to be correlated with the type of property or the year of construction.

However, the global sound quality during lockdown improved drastically, and the disturbance perceived increased, such as annoyance from neighbors. Based on the previous statement, the property’s isolation level is a crucial factor, especially when the population is forced to stay at home as a result of mobility restrictions due to COVID-19 confinement or for the new manner to work at home (telecommuting).

## Figures and Tables

**Table 1:** Correlations of the sociodemographic data.

	Age	Gender	Educational Level	Cohabitants	Modifications	Ownership	Kind of Property	Year of Construction
	*p*-Value	*p*-Value	*p*-Value	*p*-Value	*p*-Value	*p*-Value	*p*-Value	*p*-Value
Age								
Gender	<0.05							
Educational level	>0.05	>0.05						
Cohabitants	<0.01	>0.05	>0.05					
Modifications	<0.001	>0.05	<0.01	0.05				
Ownership	<0.001	>0.05	>0.05	<0.001	<0.001			
Kind of Property	>0.05	>0.05	<0.05	<0.001	<0.001	<0.001		
Year of construction	>0.05	>0.05	>0.05	>0.05	<0.001	<0.001	<0.001	
Quality of the noise before lockdown	>0.05	>0.05	>0.05	>0.05	<0.001	<0.001	<0.001	<0.001

**Table 2:** Correlations of the sociodemographic data and noise annoyance before lockdown.

Annoyance of Noise from:	Age	Gender	Educational Level	Cohabitants	Modifications	Ownership	Kind of Property	Year of Construction	Sound Quality
Outside	−0.08	0.03	0.05	−0.02	−0.02	−0.16 ***	0.24 ***	−0.18 ***	−0.65 ***
Traffic	−0.004	0.03	0.06	−0.03	0.04	−0.15 **	0.23 **	−0.12 **	−0.55 ***
Nature	0.00	−0.16 **	−0.16 **	−0.02	−0.05	0.001	−0.12 **	0.001	0.07
Neighbors	−0.13 **	−0.02	−0.05	−0.01	−0.06	−0.09 *	0.20 **	−0.072	−0.28 **
Bars, supermarkets and other business	−0.02	−0.03	0.06	0.01	0.02	−0.197 **	0.280 **	−0.176 **	−0.43 **
Trains	0.02	0.04	−0.04	−0.06	−0.05	−0.06	0.014	0.07	−0.07
Airplanes	0.78	0.21	0.821	0.44	0.357	−0.07	0.114	0.38	0.02

Significance value: * *p* < 0.05; ** *p* < 0.01; *** *p* < 0.001.

**Table 3:** Correlations of the sociodemographic data and noise annoyance during lockdown.

Annoyance of Noise from:	Age	Gender	Educational Level	Cohabitants	Modifications	Ownership	Property	Year of Construction
Outside	−0.110 **	0.047	−0.144 **	0.014	−0.06	−0.080	0.100 **	−0.090 *
Traffic	0.001	0.061	−0.024	0.010	0.024	−0.100	0.070	−0.070
Nature	−0.070	−0.190 **	−0.150 **	−0.040	−0.095	−0.11 **	0.240 ***	−0.090 *
Neighbors	−0.070	−0.193 **	−0.150 **	−0.070	−0.020	0.070	−0.130 ***	−0.001
Bars, supermarkets and other business	0.06	−0.005	−0.056	0.070	0.020	−0.140^**^	0.150 ***	−0.140 **
Trains	0.070	0.042	−0.061	−0.030	0.020	−0.050	−0.020	−0.050
Airplanes	−0.002	0.019	−0.064	0.020	−0.070	−0.030	−0.050	−0.030
Difference between phase 0 and lockdown	−0.110 **	0.092 *	0.140 **	0.050	0.040	−0.041	0.119^**^	−0.042

Significance value: * *p* < 0.05; ** *p* < 0.01; *** *p* < 0.001.

## Data Availability

In case to have access to the data, please feel free to contact the authors.
